# Obliquely Deposited Titanium Nitride Nanorod Arrays as Surface-Enhanced Raman Scattering Substrates †

**DOI:** 10.3390/s19214765

**Published:** 2019-11-02

**Authors:** Yi-Jun Jen, Meng-Jie Lin, Hou-Lon Cheang, Teh-Li Chan

**Affiliations:** Department of Electro-Optical Engineering, National Taipei University of Technology, Taipei 106, Taiwan; lmjmonty@hotmail.com (M.-J.L.); 2007library@gmail.com (H.-L.C.); derek61104@gmail.com (T.-L.C.)

**Keywords:** titanium nitride, nanorod arrays, surface-enhanced Raman scattering

## Abstract

In this work, titanium nitride (TiN) nanorod arrays were prepared as surface-enhanced Raman scattering (SERS) substrates using glancing angle deposition (GLAD) in a magnetron sputtering system. The nitrogen flow rate was varied from *R_N_*_2_ = 1 to 3 sccm, yielding five TiN uniform thin films and five TiN nanorod arrays. The figure of merit (FOM) of each TiN uniform film was measured and compared with the SERS signal of each TiN nanorod array. Rhodamine 6G (R6G) was used as the analyte in SERS measurement. For an R6G concentration of 10^−6^ M, the analytical enhancement factor (AEF) of the TiN nanorod array that was prepared at *R_N_*_2_ = 1.5 sccm was 10^4^. The time-durable SERS performance of TiN nanorod arrays was also investigated.

## 1. Introduction

Over the last several decades, surface plasmon resonance has been investigated to develop its optical and sensing applications [1,2]. When light is incident on metal nanostructures, the collective oscillation of free electrons around the nuclei in the subwavelength surface is called localized surface plasmon resonance (LSPR) [3]. The oscillation in various modes that are associated with LSPR mediates local field enhancement close to nanostructured noble metal surfaces, providing a surface enhancement effect, such as surface-enhanced Raman scattering (SERS) [4,5,6]. SERS is a powerful, nondestructive tool for ultrasensitive vibrational spectroscopy. It has been applied in chemistry and biology, including the detection of chemical and biological agents, biomedical diagnostics, DNA analysis, and pH sensing [7,8]. Most SERS performances rely on the LSPR around nm-sized gaps between metal clusters. Therefore, various metal nanostructures were developed to support the LSPR for SERS. Silver or gold nanoparticle arrays fabricated with a lithography technique have been proposed in SERS applications [9]. An alternative arrangement of metal nanostructures such as nanoparticle-on-mirror [10], metal-dielectric-metal [11] and core-shell structures [12] were reported recently to exhibit better SERS performance. An easy method to fabricate nanostructures is glancing angle deposition. Tilt nanorod arrays [13,14] and nanohelix arrays [15] have been mass-produced in a typical physical vapor deposition system and applied as SERS substrates.

The plasmonic properties for local field enhancement are strongly affected by the morphology of the nanostructure and the choice of metallic materials. Noble metals (silver and gold) are important as ideal plasmonic materials for SERS substrates [16,17,18,19]. However, they are not perfect and suffer from high cost, low stability, and poor biocompatibility. To solve these problems, semiconducting materials (TiO_2_ and ZnO) are used as SERS substrates [20,21]. Even though they exhibit local field enhancement, enhancing SERS signals, their sensitivity is relatively low. Therefore, a material with high stability and sensitivity is desired for SERS substrates.

Recently, titanium nitride (TiN) has attracted attention owing to its optical properties, which can exhibit resonant plasmon characteristics [22]. In a previous research [23], TiN has been used to exhibit localized electromagnetic field enhancements similar to those obtained using nobel metals. Additionally, TiN, with good chemical stability, a high melting point, and super hardness, becomes an important alternative plasmonic material [24]. The plasmonic properties of TiN can be varied by tuning the metal/nitrogen stoichiometry [25]. Therefore, TiN nanostructures have attracted interest when developing SERS substrates. TiN nanoparticles and semicontinuous thin films were fabricated as SERS substrates but their SERS intensities needed to be raised for real applications [26]. A semicontinuous thin film is formed in the initial stage of coating as the initially deposited grains get larger and connected [27]. In this work, TiN nanorod arrays are fabricated as SERS substrates by glancing angle deposition (GLAD) [28,29] in a magnetron sputtering system. The TiN nanorod array (NRA) is proposed to enhance stability and sensitivity for SERS applications. The TiN nanostructures have more surface area than nanoparticles, favoring molecular adhesion. The titanium/nitrogen stoichiometry can be controlled to provide optimal deposition conditions for a TiN nanorod array to generate a highly sensitive SERS signal. Since TiN exhibits high thermal and chemical stabilities, its time-durable SERS performance was also studied and compared to that of a silver nanorod array.

## 2. Experimental Setup and Methodology

**Figure 1:**
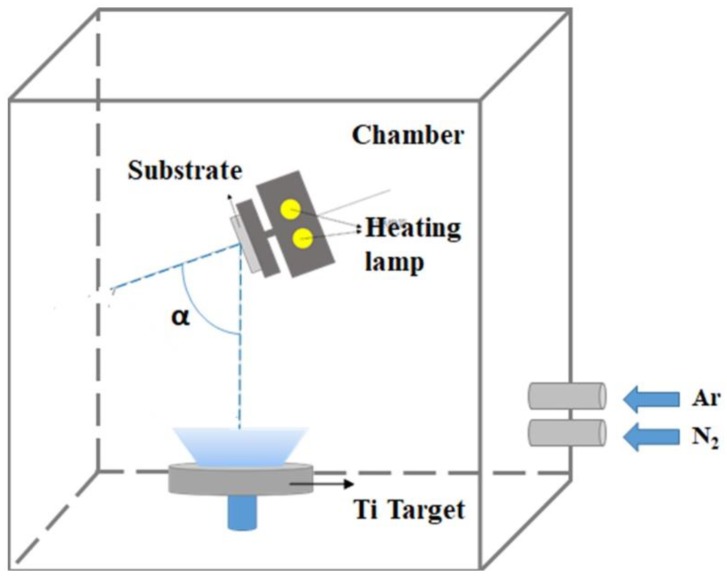
Glancing angle deposition in a magnetron sputtering system.

**Figure 2:**
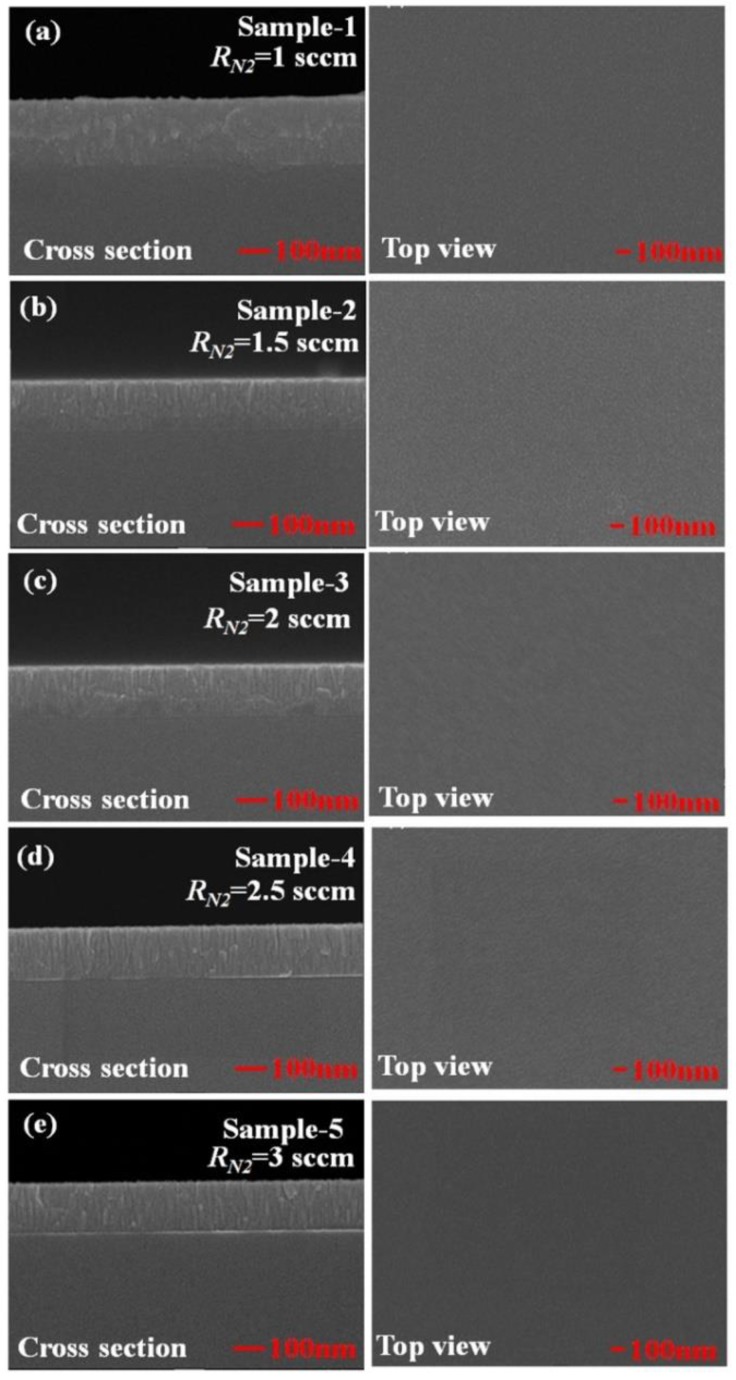
Top-view and cross-section scanning electron microscopic (SEM) images of the TiN uniform films deposited at (**a**) *R_N_*_2_ = 1 sccm, (**b**) *R_N_*_2_ = 1.5 sccm, (**c**) *R_N_*_2_ = 2 sccm, (**d**) *R_N_*_2_ = 2.5 sccm, and (**e**) *R_N_*_2_ = 3 sccm.

Both TiN nanorod arrays and uniform TiN thin films were fabricated with and without GLAD. During the initial deposition, nucleation centers randomly form on the substrate and the subsequently deposited flux causes the preferential growth of nanorods in the direction of the deposition as a result of the shadowing effect. TiN nanorod arrays were deposited on glass substrates by DC reactive magnetron sputtering (PVD Products Inc.) using a 99.995% titanium target in an argon-nitrogen environment. The base pressure in the chamber before deposition was 4 × 10^−6^ Torr. Figure 1 displays the deposition system (with a target diameter of 7.62 × 10^−2^ m), mounted at the bottom of a chamber. The substrate was mounted on a rotation stage whose center was 150 mm vertically above the target and shifted by 60 mm horizontally from the center of the target. To deposit TiN, a pure Ti target was used with Ar as the sputtering gas and N_2_ as the reactive gas. To obtain stoichiometric TiN, the argon flow was fixed at 15 standard cubic centimeter per minute (sccm) and the N_2_ flow rate was varied from *R_N_*_2_ = 1 to 3 sccm. In GLAD, the substrate is tilted at an angle α between the substrate normal and the direction of the incoming vapor flux. The deposition angle was maintained at α = 85° for TiN NRAs and α = 0° for TiN uniform thin films. Samples were deposited at an operating pressure that varied with the N_2_ flow rate. The temperature of the substrate was maintained at 200 °C during the deposition. The sputtering power was a constant 200 W (DC) in all depositions. The deposition rate was approximately 2.5 nm/min. To verify the composition of the titanium nitride, five TiN uniform films, Sample-1, Sample-2, Sample-3, Sample-4 and Sample-5, were deposited with *R_N_*_2_ = 1 sccm, *R_N_*_2_ = 1.5 sccm, *R_N_*_2_ = 2 sccm, *R_N_*_2_ = 2.5 sccm and *R_N_*_2_ = 3 sccm, respectively. Figure 2 shows top-view and cross-section scanning electron microscopic (SEM) images of the TiN uniform films. The thicknesses of the five samples were derived from the morphologies of the TiN thin films: *d*_1_ = 220 nm, *d*_2_ = 175 nm, *d*_3_ = 171 nm, *d*_4_ = 169 nm, and *d*_5_ = 166 nm. 

## 3. Results and Discussion

**Figure 3:**
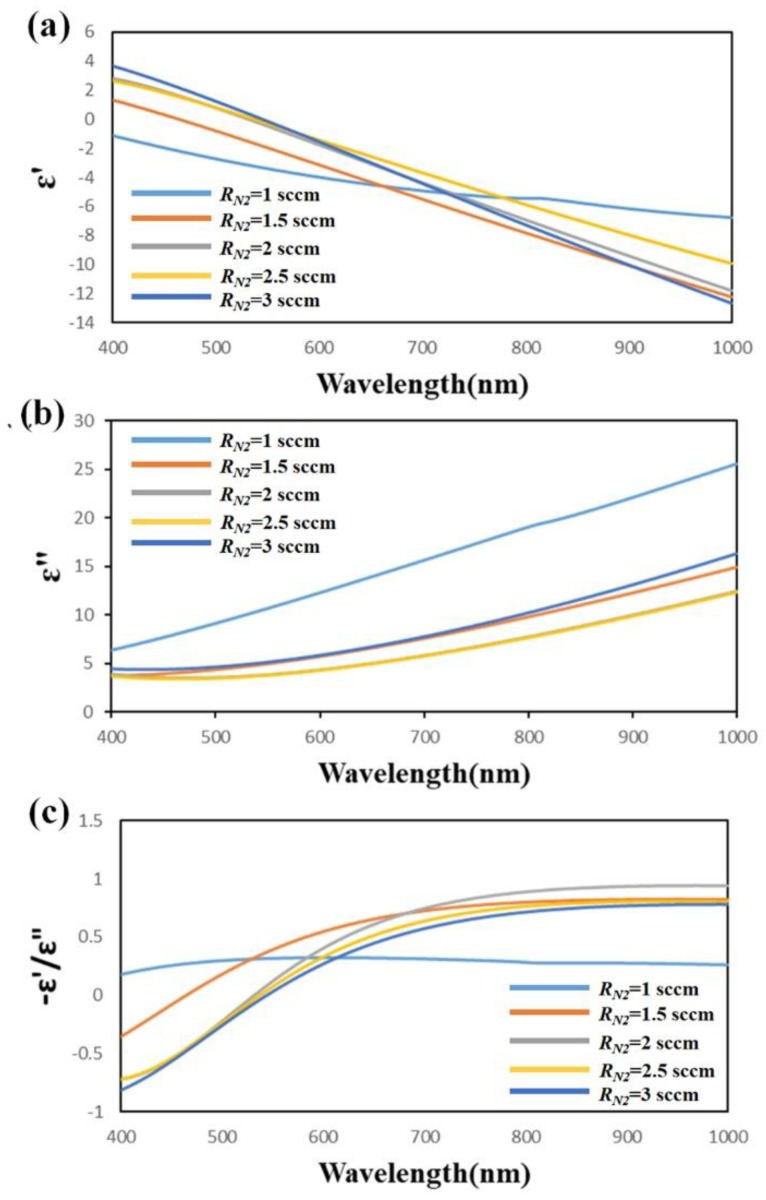
(**a**) Real and (**b**) imaginary parts of permittivity and (**c**) figure of merit (FOM) for TiN thin films grown at *R_N_*_2_ = 1 sccm, *R_N_*_2_ = 1.5 sccm, *R_N_*_2_ = 2 sccm, *R_N_*_2_ = 2.5 sccm, and *R_N_*_2_ = 3 sccm.

The optical property of TiN thin films was analyzed by variable angle spectroscopic ellipsometry (J.A. Woollam Co., VASE, Lincoln, NE, USA). The permittivity of the TiN thin films was obtained by fitting the spectroscopic elliptical parameters of the polarization state using the Drude−Lorentz model [30]. The permittivity ε = ε’ + ε” of each sample was measured as a function of wavelength, as shown in Figure 3a,b. The trend of real permittivity of the TiN thin films with *R_N_*_2_ = 1 sccm differed from that of other samples. The real part of the permittivity of Sample-1 is negative over the whole wavelengths from 400 to 1000 nm. For Sample-2 to Sample-5, the real permittivity as a function of wavelength indicates that the cross-zero wavelength λ_c_ increased from 482 to 613 nm as the flow rate of nitrogen increased from 1.5 to 3 sccm. The imaginary permittivity declined as the wavelength increased from 400 to 1000 nm for all TiN thin films. Figure 3c shows the figure of merit (FOM)—*ε*^’^*/ε*^”^, calculated from the real and imaginary parts of the permittivity for the TiN thin films with *R_N_*_2_ = 1 sccm, *R_N_*_2_ = 1.5 sccm, *R_N_*_2_ = 2 sccm, *R_N_*_2_ = 2.5 sccm, and *R_N_*_2_ = 3 sccm. The FOM is used to predict the plasmonic efficiency of plasmonic materials [31]. A higher FOM represents better plasmonic efficiency. The results reveal a maximum FOM of 0.3216 for the TiN thin film with *R_N_*_2_ = 1.5 sccm at the excitation wavelength of 532 nm.

**Figure 4:**
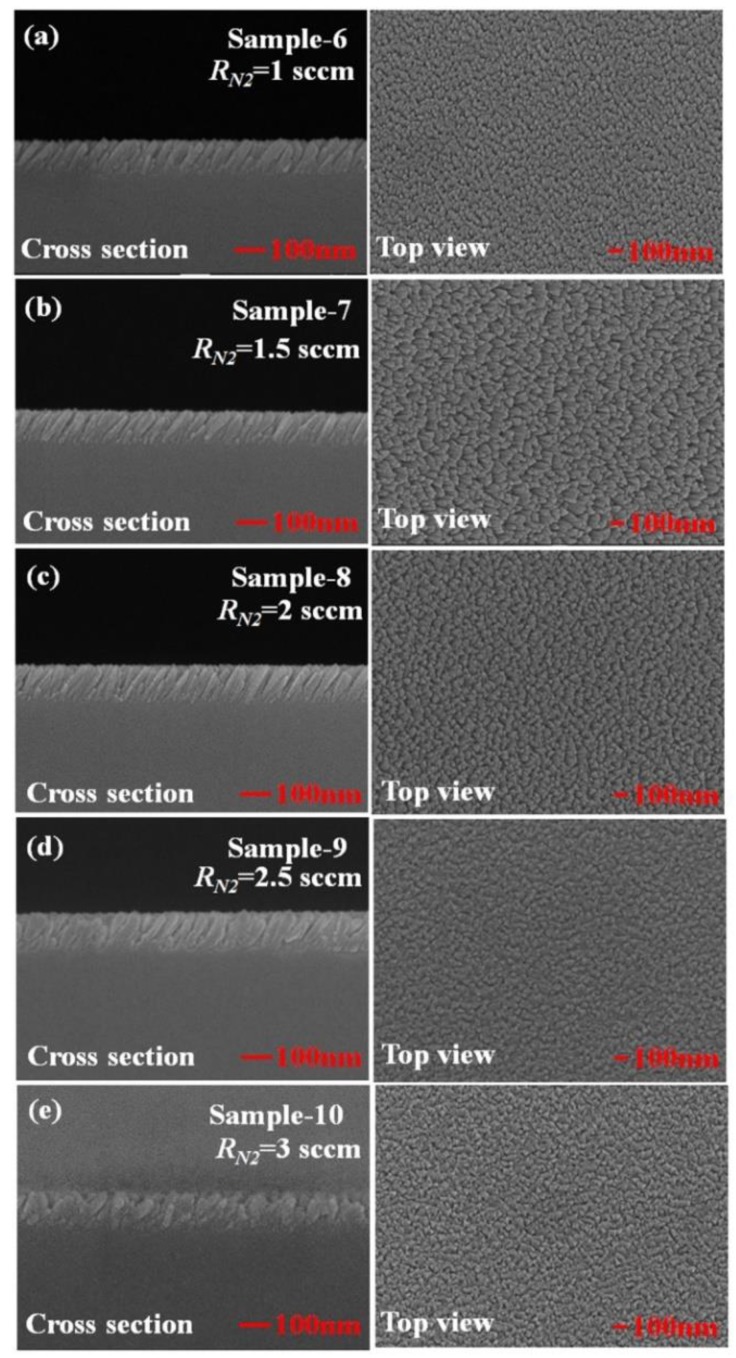
Top-view and cross-section scanning electron microscopic (SEM) images of TiN NRAs grown at (**a**) *R_N_*_2_ = 1 sccm, (**b**) *R_N_*_2_ = 1.5 sccm, (**c**) *R_N_*_2_ = 2 sccm, (**d**) *R_N_*_2_ = 2.5 sccm, and (**e**) *R_N_*_2_ = 3 sccm.

Figure 4 shows top-view and cross-section scanning electron microscopic (SEM) images of TiN NRA. Five samples, Sample-6, Sample-7, Sample-8, Sample-9, and Sample-10, were deposited using GLAD at *R_N_*_2_ = 1 sccm, *R_N_*_2_ = 1.5 sccm, *R_N_*_2_ = 2 sccm, *R_N_*_2_ = 2.5 sccm, and *R_N_*_2_ = 3 sccm, respectively. Table 1 presents the morphological parameters, including the tilt angle between the slanted rods and the surface normal *β*, the rod width *w*, the spacing between adjacent rods *p*, porosity *P*, and thickness *d*. The morphological parameters and the porosity of the TiN NRAs were estimated from the SEM images shown in Figure 4 using an image processing program (Image J). The morphological parameters in Table 1 represent the size distributions of the nanorods in the SEM images. The tilt angles ***β*** of the rods are within the range of 25° ± 5° to 39° ± 5°. The widths of the five samples vary within the range from 25 ± 5 to 36 ± 5 nm. The SEM images clearly show the form and growth direction of the TiN nanorods in Sample-6 to Sample-8. As the nitrogen flow rate increased, the form of the TiN nanorods became disordered in Sample-9 and Sample-10. In Sample-10, the growth direction of the TiN nanorods was disordered and their size was not uniform. Although α was fixed at 85°, the form and growth direction of the TiN NRAs were not similar among the five samples that were fabricated with GLAD at different nitrogen flow rates. The morphology of a TiN NRA can be controlled by varying the nitrogen flow rate.

**Figure 5:**
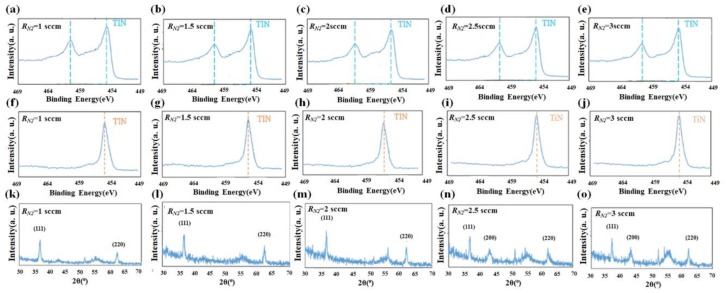
(**a**–**e**) Ti 2p and (**f**–**j**) N 1s photoemission core level spectra of TiN NRAs and (**k**–**o**) X-ray diffraction by TiN NRAs grown at *R_N_*_2_ = 1 sccm, *R_N_*_2_ = 1.5 sccm, *R_N_*_2_ = 2 sccm, *R_N_*_2_ = 2.5 sccm, and *R_N_*_2_ = 3 sccm.

TiN NRAs were analyzed by X-ray photoelectron spectroscopy (XPS). Figure 5 presents the Ti 2p and N 1s photoemission core level spectra of the five samples. The peaks in those spectra were at binding energies that corresponded to TiN [26,32]. Crystallite structures were identified by X-ray diffraction (XRD, D/MAX2500PC, Rigaku), as shown in Figure 5k–o. XRD revealed crystalline (111) and (220) in these five samples. As the nitrogen flow rate was increased, the intensity of crystalline (200) increased. The measurements are consistent with previously obtained results for crystalline TiN [32].

**Figure 6:**
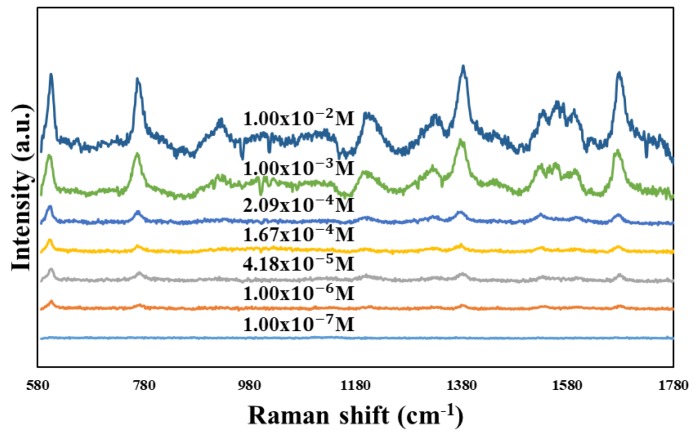
SERS spectra of R6G solution adsorbed on the TiN nanorod array for different concentrations from 10^−2^ to 10^−7^ M.

**Figure 7:**
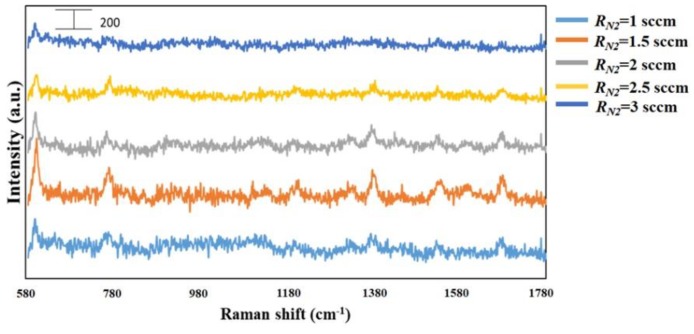
SERS spectra of TiN NRAs grown at *R_N_*_2_ = 1, 1.5, 2, 2.5, and 3 sccm.

**Figure 8:**
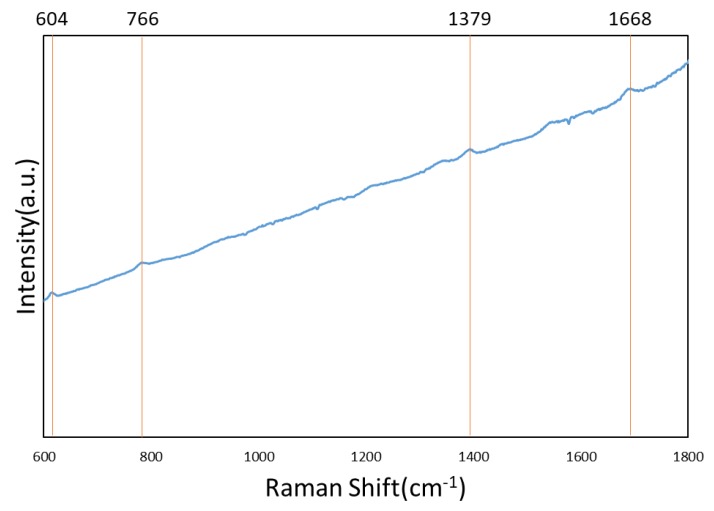
Raman spectrum of 10^−1^ M R6G adsorbed on the bare Si surface.

**Figure 9:**
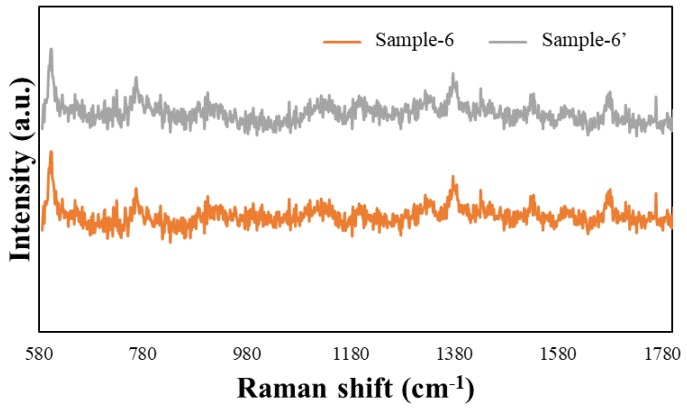
SERS spectra of Sample-6 and Sample-6′.

To perform the SERS characterization of the as-prepared TiN NRA substrates, R6G was used as a probe molecule. A 4 μL droplet of R6G methanol solution with a concentration of 10^−6^ M was dispersed on the surfaces of the TiN NRAs, which were then dried completely in air. After the droplet had evaporated, the area over which it had spread on each of the substrates was observed to be approximately 5 mm^2^. Raman spectra were obtained using a Stroker 785L Raman Spectrometer (Wasatch Photonics) with an excitation wavelength of 532 nm, power of 100 mW, a laser spot diameter of less than 50 μm, and a collection time of 30 ms. Before the saturated adsorption, the SERS signal would increase with increasing concentration of R6G molecules. Figure 6 shows the SERS intensity of Sample-7 measured at concentrations from 10^−2^ to 10^−7^ M. Figure 7 presents the SERS spectra of R6G solution that was adsorbed on Sample-6 to Sample-10. The Raman spectrum of R6G with a concentration of 10^−1^ M, absorbed on the bare Si surface was obtained as a reference, as shown in Figure 8. The characteristic Raman vibrational peaks of R6G can be well resolved at 604, 766, 1379, and 1668 cm^−1^. The Raman bands at 1688, 1379, 766, and 604 cm^−1^ are attributed to xanthene ring stretching, ethylamine group wagging, and carbon–oxygen stretching [33]. The noise spectra exhibit none of the spectral features associated with R6G. The signal-to-noise peaks at specific bands represent the characteristic of R6G [34]. The intensities of the Raman peaks of R6G for the TiN NRA deposited at *R_N_*_2_ = 1.5 sccm exceed those of other TiN NRAs. The characteristic Raman peak intensity of R6G at 604 cm^−1^ is greatest for the TiN NRAs. The variations of Raman peak intensities for samples deposited under the same condition are less than 10%. The SERS spectra of two samples (Sample-6 and Sample-6′) deposited at *R_N_*_2_ = 1.0 sccm are shown in Figure 9 to present the repetition in our experiment. 

To compare the enhancements of the TiN NRAs, the analytical enhancement factor (AEF) was calculated using the following formula [35].

$$\mathrm{AEF}=\frac{I_{\mathrm{SERS}}/C_{\mathrm{SERS}}}{I_{\mathrm{Ref}}/C_{\mathrm{Ref}}},$$

where I_SERS_ and C_SERS_ are the intensity of the SERS spectrum and the concentration of R6G molecules that were adsorbed on the SERS substrate, respectively, and I_Ref_ and C_Ref_ are the intensity of the non-SERS spectrum and the concentration of R6G that was adsorbed on the reference substrate. Table 2 presents the AEF of TiN NRAs for an R6G concentration of 10^−6^ M. The largest AEF value of the characteristic Raman peak intensity of R6G at 604 cm^−1^ is 1.34 × 10^4^, obtained from Sample-7. As the N_2_ flow rate increases from 1.5 to 3 sccm, the AEF decreases. Sample-7 exhibits more SERS enhancement than other samples. The AEF results are consistent with the FOM of the permittivity for TiN NRAs.

The main factor that influences the SERS performance is the permittivity of TiN. The permittivity depends on the deposition parameter including substrate temperature, gas flow, and direction of vapor flux. From Figure 4a, the real part of permittivity becomes negative for wavelengths larger than the cross-zero wavelength. The magnitude of negative permittivity is largest for the *N_2_* flow rate of 1.5 sccm, which leads to a metal-like property of TiN. On the other hand, the FOM shown in Figure 4c also indicates that the sample deposited at *R_N_*_2_ = 1.5 sccm has the highest FOM at wavelengths between 530 and 678 nm. The SERS performance is compared with two previous works of using TiN nanostructures as SERS substrates. The SERS AEF measured from a TiN thin film prepared via nitridation of sol-gel derived TiO_2_ film is less than 10^4^ for a R6G concentration of 10^−6^ M [36]. The similar AEF less than 10^4^ for the same R6G concentration was also measured from TiN nanorod arrays that were prepared using a hydrothermal process followed by nitridation in ammonia atmosphere [37].

**Figure 10:**
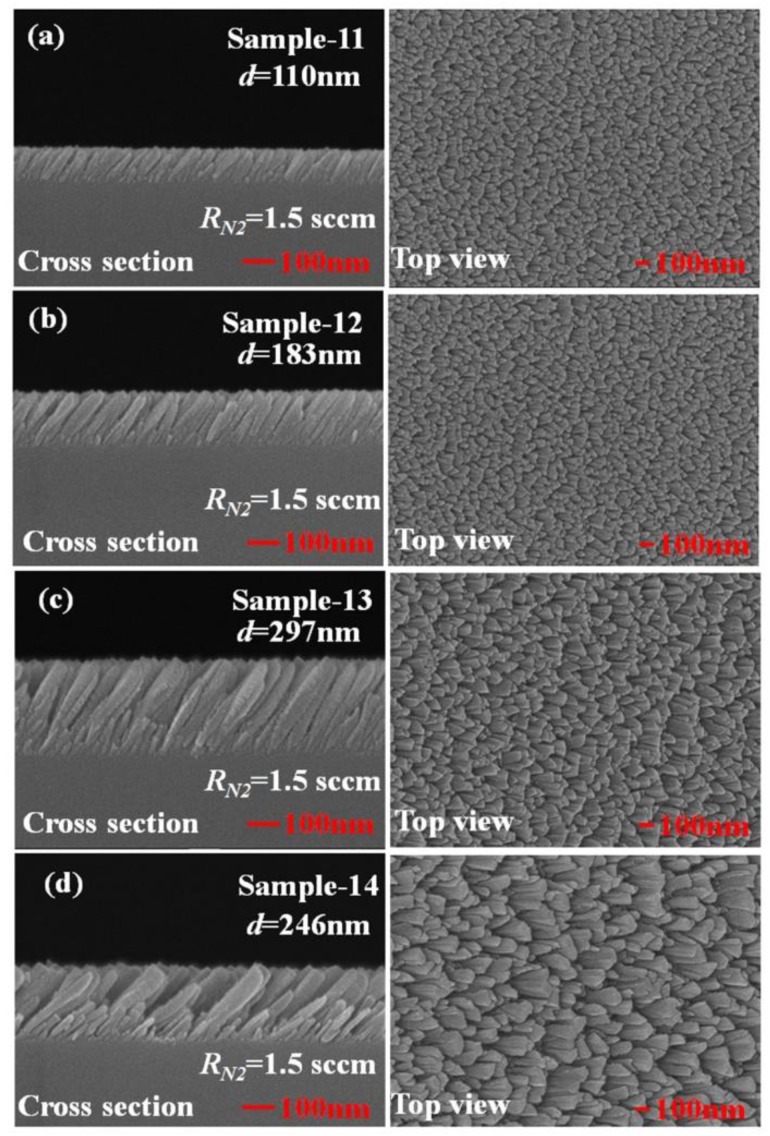
Top-view and cross-section scanning electron microscopic (SEM) images of TiN NRAs with thickness d = (**a**) 110 nm, (**b**) 183 nm, (**c**) 297 nm, and (**d**) 246 nm.

**Figure 11:**
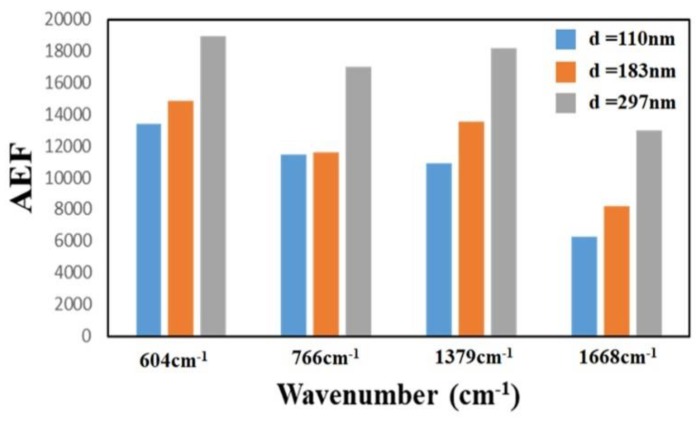
AEF for Sample-11 (*d* = 110 nm), Sample-12 (*d* = 183 nm) and Sample-13 (*d* = 297 nm) at 604 cm^−1^.

**Figure 12:**
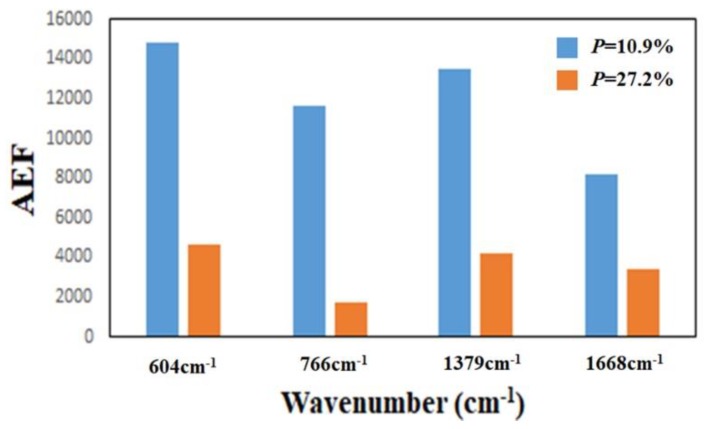
AEF for Sample-12 (*P* = 10.9%) and Sample-14 (*P* = 27.2%) at 604 cm^−1^.

To investigate the effect of the thickness of the TiN NRAs on the Raman enhancement therein, Sample-11, Ssmple-12, and Sample-13 were fabricated at α = 85° with *R_N_*_2_ = 1.5 sccm. Table 3 presents the morphological parameters, including average tilt angle between the slanted rod and the surface normal β, the rod width *w*, the spacing between adjacent rods *p*, porosity, and thickness *d*. Figure 10a–c show the morphologies of Sample-11, Sample-12 and Sample-13 with thicknesses of 110 ± 5, 183 ± 10, and 297 ± 15 nm, respectively. As the thickness increased from 110 ± 5 to 297 ± 15 nm, the mean width of the nanorods increased from 26 to 58 nm. *The competitive* growth of the TiN nanorods during *glancing angle deposition* influences the morphology of a TiN NRA. The *fan-out* phenomenon of nanorods is caused by surface diffusion of particles as the thickness increases. Figure 11 shows the AEF of Sample-11, Sample-12, and Sample-13 with an R6G concentration of 10^−6^ M. The largest AEF is 1.89 × 10^4^ for Sample-13 at 604 cm^−1^. The AEF values increase with thickness at 604, 766, 1379, and 1668 cm^−1^ because longer TiN nanorods own larger surface areas to adsorb molecules. The adsorbed molecules enhance the probability of Raman scattering. Sample-14 was fabricated at α = 85° and *R_N_*_2_ = 1.5 sccm to elucidate the effect of the porosity of the TiN NRA by limiting the direction of the deposition flux. The flux was collimated using a plate 10 mm above and parallel to the substrate, to limit its distribution and thereby control the porosity [38]. A comparison of Sample-14 with Sample-12 indicates that the collimation increased the average tilt angle of the rods from 32° ± 5° to 40° ± 3°and the porosity from 10.9% to 27.2%. Figure 12 shows the AEFs of Sample-12 and Sample-14 at 604 cm^−1^ for an R6G concentration of 10^−6^ M. Although Sample-14 is thicker than Sample-12, the largest AEF value, 1.48 × 10^4^, is obtained for Sample-12 at 604 cm^−1^. The narrow spaces among TiN nanorods in Sample-12 with low porosity generate strong local electric fields in the gaps [39], enhancing Raman scattering. 

**Figure 13:**
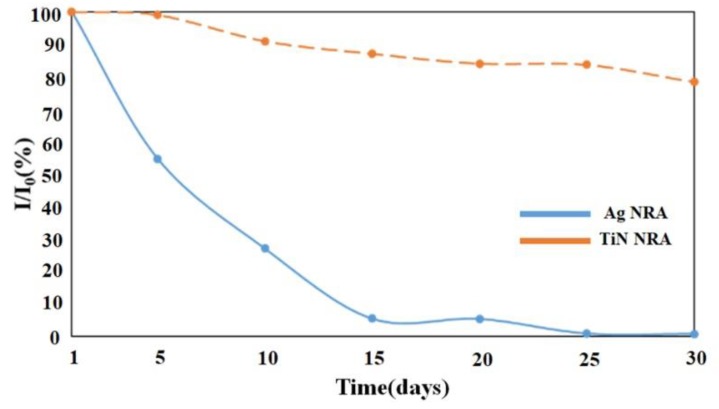
Variation of Raman peak intensity at 604 cm^−1^ for the Ag and TiN NRAs.

The stability of the TiN NRA was investigated. The SERS enhancement of the TiN NRA was measured and compared with those of a slanted Ag nanorod array (NRA) that had been previously developed as a highly sensitive SERS substrate [23]. The Ag NRA with a thickness of 217 nm was obliquely deposited by electron beam evaporation at a deposition angle of 88°. Figure 13 shows the variation of Raman peak intensity over time at 604 cm^−1^ for the Ag and TiN NRAs, respectively. After 15 days, the SERS enhancement of the Ag NRA was reduced to 6% relative to that of fresh Ag NRA after coating. The SERS enhancement of the TiN NRA remains above 90% for 10 days. After 30 days, the SERS enhancement of the TiN NRA is reduced by only 22%. The high stability of the Raman spectrum indicates the potential SERS application of the TiN NRAs.

## 4. Conclusions

TiN NRAs were successfully fabricated as SERS substrates by glancing angle deposition in a DC reactive magnetron sputtering system. The morphology of a TiN NRA can be controlled by tuning the N_2_ flow rate from *R_N_*_2_ = 1 to 3 sccm. For a fixed deposition angle of 85° and similar thicknesses around 120 nm, the optimal nitrogen flow rate is *R_N_*_2_ = 1.5 sccm, which provides the strongest SERS signal from a TiN nanorod array and the highest FOM of a uniform TiN thin film. The highest AEF is 10^4^ at 604 cm^−1^ for R6G with a concentration of $10^{-6}$M. The SERS signal gradually increases with thickness. At a thickness of 297 nm, the AEF is 1.89 × 10^4^. The SERS enhancement of TiN NRA remains above 78% in air for 30 days. Based on this work, the TiN NRA as a SERS substrate can be mass-produced using GLAD and it is sufficiently durable for use in harsh environments.

## Figures and Tables

**Table 1:** Average tilt angle (β), rod width (w), spacing between adjacent rods (p), porosity (P), and thickness (d) of TiN NRA.

	β (°)	w (nm)	d (nm)	p (nm)	P (%)
Sample-6	39 ± 5	30 ± 5	117 ± 10	21 ± 5	7.4
Sample-7	32 ± 5	26 ± 5	112 ± 15	30 ± 5	10.3
Sample-8	25 ± 5	25 ± 5	123 ± 15	21 ± 5	8.3
Sample-9	31 ± 5	28 ± 5	130 ± 8	22 ± 5	9.5
Sample-10	37 ± 5	36 ± 5	134 ± 6	20 ± 5	8.9

**Table 2:** Analytical enhancement factor (AEF) of the TiN NRA with an R6G concentration of 10^−6^ M.

Raman Peak (cm−1)	Sample-6 AEF	Sample-7 AEF	Sample-8 AEF	Sample-9 AEF	Sample-10 AEF
604	8.39 × 103	1.34 × 104	7.59 × 103	6.53 × 103	4.98 × 103
766	3.01 × 103	1.15 × 104	2.42 × 103	4.05 × 103	3.60 × 103
1379	4.94 × 103	1.09 × 104	4.47 × 103	5.08 × 103	4.81 × 103
1668	3.70 × 103	6.26 × 103	1.90 × 103	2.09 × 103	3.90 × 103

**Table 3:** Tilt angle (β), rod width (w), spacing between adjacent rods (p), porosity (P), and thickness (d) of TiN NRA.

	β (°)	w (nm)	d (nm)	p (nm)	P (%)
Sample-11	31 ± 5	26 ± 5	110 ± 5	30 ± 5	9.3
Sample-12	32 ± 5	33 ± 3	183 ± 10	34 ± 5	10.9
Sample-13	35 ± 5	58 ± 5	297 ± 15	71 ± 15	17.5
Sample-14	40 ± 3	48 ± 5	246 ± 15	159 ± 20	27.2
